# Participatory evaluation of delivery of animal health care services by community animal health workers in Karamoja region of Uganda

**DOI:** 10.1371/journal.pone.0179110

**Published:** 2017-06-08

**Authors:** James Bugeza, Clovice Kankya, James Muleme, Ann Akandinda, Joseph Sserugga, Noelina Nantima, Edward Okori, Terence Odoch

**Affiliations:** 1 Department of Livestock Health, National Livestock Resources Research Institute (NaLIRRI), Tororo, Uganda; 2 Participatory Epidemiology Network in Uganda (PENU), Wandegeya, Kampala, Uganda; 3 Department of Biosecurity Ecosystems and Veterinary Public Health-College of Veterinary Medicine, Animal Resources and Biosecurity, Makerere University, Kampala, Uganda; 4 Department of Livestock Health and Entomology, Ministry of Agriculture Animal Industry and Fisheries, Entebbe, Uganda; 5 Food and Agriculture Organization of the United Nations, Department of Livestock Health, Wandegeya, Kampala, Uganda; International Nutrition Inc, UNITED STATES

## Abstract

**Aim:**

An evaluation exercise was carried out to assess the performance of Community Animal Health Workers (CAHWs) in the delivery of animal health care services in Karamoja region, identify capacity gaps and recommend remedial measures.

**Materials & methods:**

Participatory methods were used to design data collection tools. Questionnaires were administered to 204 CAHWs, 215 farmers and 7 District Veterinary Officers (DVOs) to collect quantitative data. Seven DVOs and 1 Non Government Organization (NGO) representative were interviewed as key informants and one focus group discussion was conducted with a farmer group in Nakapiripirit to collect qualitative data. Questionnaire data was analyzed using SPSS version 19. Key messages from interviews and the focus group discussion were recorded in a notebook and reported verbatim.

**Results:**

70% of the farmers revealed that CAHWs are the most readily available animal health care service providers in their respective villages. CAHWs were instrumental in treatment of sick animals, disease surveillance, control of external parasites, animal production, vaccination, reporting, animal identification, and performing minor surgeries. Regarding their overall performance 88.8%(191/215) of the farmers said they were impressed. The main challenges faced by the CAHWs were inadequate facilitation, lack of tools and equipments, unwillingness of government to integrate them into the formal extension system, poor information flow, limited technical capacity to diagnose diseases, unwillingness of farmers to pay for services and sustainability issues.

**Conclusions and recommendations:**

CAHWs remain the main source of animal health care services in Karamoja region and their services are largely satisfactory. The technical deficits identified require continuous capacity building programs, close supervision and technical backstopping. For sustainability of animal health care services in the region continuous training and strategic deployment of paraprofessionals that are formally recognised by the traditional civil service to gradually replace CAHWs is recommended.

## Introduction

Livestock and livestock products play a key role in raising incomes of households and providing a source of protein to many families. Indeed, according to analysis of poverty trends using the Uganda National Household Survey (UNHS), households that have a crop livestock enterprise mix tend to be generally less poor [1]. According to Uganda Bureau of Statistics UBOS [2],Uganda has an estimated 14 million cattle, 4 million sheep, and 14 million goats, most of which are kept in the semi-arid cattle corridor. However, delivery of veterinary services in areas of low population density, particularly where there has been experience of conflict or insecurity, and where there may be diverse and nomadic populations, can pose significant challenges. One such obstacle is the high prevalence of hard-to-reach populations living in physically remote areas. More fundamentally, in many of these contexts the state is either absent or perceived as remote, and this in turn can reinforce a sense of mistrust and undermine state–community relations [3] [4]. Indeed, there has been a gradual decline in the delivery of state veterinary services due to unfavorable policies and insecurity in some parts of Uganda, especially the Karamoja region. Diseases like Foot and Mouth Disease (FMD), Contagious Bovine Pleuropneumonia (CBPP), Peste des Petits Ruminants (PPR), Contagious Caprine Pleuropneumonia (CCPP), East Coast Fever (ECF) and others continue to cause serious socio-economic consequences including production losses, loss of livelihoods, poverty, food insecurity, restriction of marketing opportunities, disincentives to investment and public-health risks. The most vulnerable groups, for whom animal diseases are particularly devastating, are poor livestock farmers and farming communities [5].

As part of the structural adjustment programs of the International Monetary Fund (IMF) and World Bank in the late 1980s, the government of Uganda liberalized and decentralized the provision of veterinary services just like in other parts of Africa, such as Ethiopia and Somalia. As a result, many actors became involved in the provision of veterinary services in Uganda [6]. The provision of clinical services, breeding and spraying for tick control were privatized, while vaccination of animals against epidemic diseases, quarantines and tsetse control were retained under the Ministry of Agriculture, Animal Industry and Fisheries [6]. It is important to note, however, that private veterinary services were concentrated largely in urban and peri-urban areas that have favorable infrastructure and a high potential for business [7]. The often remote livestock-keeping areas still largely depend on the state veterinary services, that are lacking in many aspects; moreover, few veterinarians are willing to accept the hostile living conditions there [7]. Under such circumstances the CAHWs system has been promoted as an alternative solution to providing animal health services in marginal, often hard-to-reach areas [4]. Experiences across Africa have shown that community based animal health workers can provide effective animal health care services for pastoral communities that are often highly mobile [8,9]. Presently, community-based animal health systems cover extensive areas of pastoral northern Kenya, northeastern Uganda, the Afar region of Ethiopia and northern Tanzania [10].

In Uganda, CAHWs have been instrumental in the delivery of primary animal health care services in the Karamoja region. Karamoja region is predominantly inhabited by pastoral and agro-pastoral groups that share common languages, culture, history and livelihood systems across northeastern Uganda, northwestern Kenya, southeastern South Sudan and southwestern Ethiopia [11]. Karamoja is a semi-arid region characterized by low level, erratic rainfall patterns and is considered marginal. The region presents a unique socio-economic and cultural background that requires a unique interventional approach, necessary for meeting the livestock development needs. The dominating livelihood activities are pastoralism and agro-pastoralism with a focus on livestock production [11]. It is believed that 15 percent of household food energy consumption comes from milk and milk products. Thus, the increasing threat from outbreaks of livestock diseases, notably PPR, FMD, CBPP, CCPP and Sheep/Goat Pox, among others, against a backdrop of inadequate public veterinary services is threatening not only the livestock population but, to a large extent, the livelihoods in the region. The use of CAHWs in Karamoja started in 1994 with the Pan-African Rinderpest Campaign (PARC). Subsequently, other NGOs such as Christian Veterinary Mission (CVM), Church of Uganda Livestock Extension Program (LEP), Christian International Peace Services (CHIPs), Lutheran World Federation (LWF), Oxfam and Food and Agriculture Organization (FAO) sponsored trainings of CAHWS, in addition to the development of a harmonized users’ manual for all CAHWs. The CAHWs are meant to provide primary animal health-care services at community level, and they are linked to a drug supply system, as well as a referral system with veterinary professionals and the District Veterinary Officers (DVOs). In this way they complement the government veterinary extension system that is severely constrained and overstretched [8].

However, to date, no studies have been conducted to assess the impact the CAHWs have had on the delivery of animal health care services and to identify factors that have contributed to their success or failure in Uganda. This study examines the effectiveness of the CAHWs in the delivery of primary animal health care services in Karamoja region of Uganda. The evaluation exercise was carried out between July and September 2016. The aim was to identify capacity gaps and recommend remedial actions.

## Materials and methods

### Study area

The evaluation was carried out in the 7 districts that make up the Karamoja region of Uganda. This area constitutes 33% of Uganda’s rangelands, 16% of the human population and 25% of its livestock [12]. The inhabitants of Karamoja, known collectively and generically as the Karamojong, are made up of three main ethnicities; namely the Dodoso, Jie, and Karimojong, the latter of which are subdivided into the Bokora, Matheniko, and Pian. Along the border with Kenya to the east are the Turkana and Pokot tribes, while to the north in Sudan are the Toposa. The so-called “Karamoja cluster” extends from these shared borderlands of Uganda, Kenya, and Sudan to the southwestern corner of Ethiopia. Most Karamojong practice agro-pastoralism across the semi-arid and arid plains of this region, although in Karamoja there is sufficient ecological variation to constitute three distinct production zones; namely agricultural, agro-pastoral and pastoral [13]. The agricultural zone mostly runs along the western border of Karamoja, and is also referred to as the “green belt” of Karamoja. In the green belt, where rainfall, on average, is nearly double that of the pastoral areas, a wide variety of crops can be grown, including corn, sorghum, beans, millet, cow peas, ground nuts, and a number of tropical fruits [13]. However, the majority of Karamojong live in the agro-pastoral and pastoral areas, and livelihoods there are based primarily on livestock rearing.

For hundreds of years, the arid and drought-prone nature of much of Karamoja has made food security and group survival often difficult and precarious. Cattle are highly valued, not only as a means of providing sustenance but also as bride wealth, social status, and a ceremonial centerpiece [12]

### Study design

A mixed methods design, where participatory approaches were used to collect both qualitative and quantitative data using a mixture of tools, was implemented. Quantitative data was collected using questionnaires, and qualitative data was obtained using interview guides and checklists, during key informant interviews and focus group discussions, respectively.

### Designing of evaluation tools

Participatory approaches described by Catley and Mariner [7], Barahona and Levy [14] and Allepuz et al. [15] were used to develop the evaluation tools. In consultation with the DVOs in the region, key stakeholders to be involved in developing evaluation tools were identified. The key stakeholders identified were; Community Animal Health Workers, veterinarians both public and private, NGO representatives from FAO, OXFAM, Jie Community Animal Health Workers Association (JICAHWA); Political leaders, farmers’ representatives and representatives of Community Based Organizations (CBOs). Three stakeholder consultation workshops were conducted in Abim, Moroto and Kotido districts to draft the evaluation tools. Participatory methods including meetings, focus group discussions (FGDs), key informant interviews, metaplan and problem tree were used to identify key functions of CAHWs that could be assessed, the best way to assess them and the type of questions that could be asked in order to obtain answers that would be used to assess a particular function.

The Metaplan method [16] was used to identify the key functions of CAHWs. Each participant was given 3 manila cards and asked to write only one function of the CAHWs on each card.

The facilitator collected the cards afterwards, the participants gathered in a semicircle in an open area and functions of the CAHWs identified were read out one by one by the facilitator and time was allowed for the participants to discuss each of the functions as read out until consensus was obtained. After discussions and agreement among the participants, the functions of CAHWs were categorized and those falling within a given category grouped together. The functions agreed upon in this way were then noted down.

The problem tree method was used identify problems faced by the CAHWs, the root causes and the consequences of this problem on the performance of their functions. The stem represented the problem, the roots represented the causes and the branches represented the consequences. Participants were provided with cards and asked to write one key challenge per card. The cards were collected by the facilitator and read out to the participants and after agreement among themselves challenges falling in the same category were grouped together. The facilitators then led the participants in identifying the causes and consequences of each problem in a plenary.

Focus group discussions were then held to develop a criteria grid (S1 Table). During this session, participants were divided into 4 groups, each comprising of a veterinarian, CAHWs, NGO representatives and other stakeholders. The facilitators ensured a good mix of the participants. Each group was allocated two functions (earlier identified in stage 1), and for each function they were tasked to identify the best ways of performing the functions, respondents to be interviewed to assess the performance of the functions and to suggest possible questions that could be asked during data collection to assess the function. Using information from the criteria grid (S1 Table), different tools were designed by the research team for collecting data from CAHWs, farmers, DVOs and NGO representatives.

A one-day consultative workshop was finally held in Moroto to validate and further refine the evaluation tools. The workshop was attended by DVOs from all the 7 districts, selected CAHWs and NGO representatives. After this phase, the refined tools were ready for pre-testing. For pre-testing the tools, DVOs nominated research assistants who were trained by PENU consultants, and thereafter pre-testing was done jointly.

### Sample size determination and sampling of respondents

Being a hard-to-reach area, certain considerations were made in determining the number of CAHWs and farmers to interview. Some of the considerations included location, accessibility, availability of a CAHW in a given locality, security, distances involved, number of Manyatas (collection of huts representing a homestead) in a given area, and number of days available to execute the work. Accordingly a total of 215 farmers and 204 CAHWs were purposively selected for this exercise. All the 7 DVOs were also purposively selected to fill out a questionnaire. In addition, all 7 DVOs, plus one NGO representative were selected for the key informant interviews, and one focus group was chosen for additional discussions. The focus group was purposively chosen, because the leader of the group in Namalu sub county, Nakapiripirit district accepted to mobilize the members on the agreed date.

### Data collection

Trained research assistants collected quantitative data from farmers and CAHWs using structured questionnaires (S1 and S2 Texts) respectively in their domiciles and the DVOs played the supervisory role. All the research assistants were natives who could ably speak the native dialects.

To ensure confidentiality of data each respondent was assigned a unique identification number, and as such, their names were not required on the questionnaire forms. Ethical clearance was not sought from a requisite institutional review board (IRB) prior to this evaluation exercise, as this was considered a baseline survey to gather evidence for future interventions. Nevertheless, oral consent (literacy problems in the area complicated obtaining written consent) was obtained from the participants before questionnaires were administered. Each of the 7 DVOs filled out their questionnaire (S4 Text). PENU consultants collected qualitative data from key informants (DVOs and NGO representative) using key informant interview guide (S1 Text). The consultants also collected additional information from the focus group discussion conducted at Namalu using a FGD guide (S1 Text).

### Data management and analysis

Questionnaires were checked for completeness and consistency. Completed questionnaires were entered in Microsoft Excel, cleaned and checked for missing values using the filter function. Quantitative data were analyzed using the SPSS version 19. Descriptive statistics were calculated and results presented in tables and graphs. To assess the technical ability of CAHWs with regard to treatment of diseases (S3 Text, Questions 2 and 3), a single suitable answer to any of those questions meant that the CAHW had an idea and this was considered in classifying his/her response as correct or incorrect. However, to validate the field assessment, the questionnaires were given to two independent veterinarians to mark. The results were compared afterwards and the research team classified the responses accordingly. Qualitative data was reported verbatim.

## Results

### Problems, causes and consequences to the performance of CAHWs

The list of problems faced by CAHWs in the execution of their duties, their causes and consequences are presented in Table 1 below

**Table 1:** Problems, causes and consequences to the performance of CAHWs.

Problems/challenges	Main causes	Consequences
Poor information flow	• Lack of a common voice by the CAHWs association to air out their views • CAHWs association in Abim is inactive • Conflicting information from partners/authorities • Lack of coordination between CAHWs	• Morale to perform duties is low • Failure to perform activities • Lose support from development partners • Dissolution of leadership
Old tools and equipment	• Over use • Lack of spare parts • Delayed replacement • Lack of stockists for the equipment in the region • Very expensive for CAHW to afford	• Improper treatment (over-dosing or under-dosing of animals) • Inaccurate diagnosis • Causes injuries to animals e.g. de-horning using old tools • Failure to perform the required surgical procedure • Farmers lose confidence and trust in CAHWs
Lack of transport means	• Farmers have moved to re-settlement areas which are far • Bad weather, especially during rainy seasons • Old bicycles • Poor access roads	• Failure or delayed response to disease cases • Late treatment of animals, leading to death • Difficulties in data collection
Delay in reporting diseases	• Farmers try to minimize the cost of treatment by attempting to treat their own animals • Farmers try indigenous solutions to disease problems first • Difficulty in accessing CAHWs by farmers	• Death of animals • CAHWs lose jobs • Diseases continue to spread to other animals • Drugs expire • Loss of income
Farmers not willing to pay	• Overcharging farmers by CAHWs • Farmers not aware of cost of some drugs e.g. Parvexon, Imizol • Farmers not aware that services of CAHWs are private and supposed to be paid for • Poor approach to farmers by CAHW • Farmers under look (do not appreciate) the services of CAHWs • Farmers attitude towards service delivery i.e. they think that services are paid for by the government • Political pro-announcements that services are free • Government has not clarified on ‘Private good’ diseases • Misuse of drugs by farmers • Farmers are still relief minded • Lack of exit strategy by NGOs • Lack of information sharing between political leaders, farmers and CAHWs • Farmers do not look at animal rearing as a business	• No follow up for the treated animals • Death of animals • Some drugs will expire • CAHWs become inactive and sell their equipments • CAHWs do not practice and hence loose skills • Loss of morale to offer services • Sustainability of services is affected • Farmers lose trust in government vets and CAHWs • Confusion between free services and what needs to be paid for
Failure by CAHWs to properly identify diseases	• Shorter times for trainings • Mixed infections • Lack of laboratories for testing blood • Lack of support supervision • Limited skills • No standardized curriculum for training	• Disease out breaks • Wrong medication • Wrong reporting samples • Wrong medication • Wrong decisions taken • Death of animals

### The criteria grid

The criteria grid (S1 Table), developed jointly with various stakeholders, formed the basis of developing the data capture tools for farmers, CAHWs, DVOs, NGOs and focus groups.

### Functions of CAHWs

Overall, 8 functions of CAHWs were identified and assessed in this evaluation. The functions were; Treatment, Disease surveillance, control of external parasites, Animal production, vaccination, reporting, animal identification, and performing minor surgeries such as dehorning and castration.

### Summary statistics of the respondents

Table 2 below shows the number of respondents interviewed per district

**Table 2:** Number of respondents per district.

District	Category		Percentage	
	CAHWs	Farmers	CAHWs	Farmers
Napak	29	32	14.2	14.9
Nakapiripirit	30	30	14.7	14.0
Amudat	28	31	13.7	14.4
Moroto	30	30	14.7	14.0
Kaabong	27	31	13.2	14.4
Kotido	30	31	14.7	14.4
Abim	30	30	14.7	14.0
**Total**	**204**	**215**	**100.0**	**100.0**

A total of 204 CAHWs and 215 farmers were interviewed from the 7 districts with near equal representation

### Treatment, record keeping and technical ability of the CAHWs

Results of the assessment of performance of the function of treatment and record keeping are shown in S2 Table. Tick borne diseases (TBD) were the commonest diseases mentioned by CAHWs. The main TBDs were Anaplasmosis and East Coast Fever; as mentioned by 75.5% (154/204) and 53.4% (109/204) of the CAHWs, respectively. Respiratory diseases, like Contagious Pleuropneumonia (CBPP) and Contagious Caprine Pleuropneumonia (CCPP), were also quite common, mentioned by 70.6% (144/204) and 35.3% (72/204) of the CAHWs, respectively. These findings were corroborated by comments from DVOs, who mentioned that CBPP, PPR, Newcastle disease (NCD), Lumpy Skin Disease (LSD), Foot and Mouth Disease (FMD), CCPP and African Swine Fever (ASF) disease outbreaks have been experienced in the past 5 years.

Key informants reported, *“Brucellosis is top on the list of zoonotic diseases in Kaabong district leading to reduced livestock productivity and a threat to the health of farmers*.*”(DVO Kaabong district)*. However, the evaluation revealed that only 10.8% (22/204) of the CAHWs reported brucellosis as a common disease in the area.

On whether CAHWs could diagnose any 3 livestock diseases of their choice, 91.2% (186/204) could correctly diagnose their chosen disease 1, 81.9% (167/204) for their second choice of disease and 83.3% (170/204) for their third disease, based on clinical signs. Similarly, most of the CAHWs could ably prescribe effective treatment for the diseases of their choice; as indicated by 84.4% (173/204) for disease 1, 78.9% (161/204) for disease 2 and 77.9% (159/204) for disease 3. These findings also agree with comments from DVOs who expressed satisfaction about the CAHWs role in treatment of sick animals and agreed that they (CAHWs) promptly report diseases in 12–24 hours. Additionally, DVOs agreed that CAHWs have received refresher trainings regarding disease diagnosis and treatment, which further augments their technical capabilities.

On the availability of CAHWs when needed to attend to sick animals, most farmers interviewed (79.5%; 171/215) indicated that CAHWs play a very important role in the provision of animal health care services. *“For one to qualify to be a CAHW*, *the level of activity of someone in the community is very key*, *although other aspects like being a resident of that particular community are considered”*, reported most DVOs.

Results from 7.4% (16/215) of the farmers indicated that traditional healers play roles in provision of animal health care services, an indicator of the importance of ethno-veterinary aspects where conventional approaches are inadequate. The majority (69.8%; 150/215) of farmers revealed that CAHWs are the most readily available animal health care service providers in their respective villages. In addition, the CAHWs do respond to farmers’ calls most of the time whenever requested. Above half (56.7%; 122/215) of the farmers indicated that, whenever called upon, the CAHWs take 12 hours or less to respond to the call.

Most farmers rated CAHWs’ technical ability and performance as impressive. This was validated by the statements made by the majority of the DVOs during the evaluation exercise, as follows; *“As DVOs*, *our role is to ensure that all CAHWs are trained on several aspects of animal diseases*. *Here*, *our CAHWs receive trainings on disease surveillance and reporting*, *disease diagnosis and treatment*, *records keeping*, *vaccine handling and vaccination*, *meat inspection and public health*, *animal husbandry practices”*. However, results (S2 Table) of the opinion of the DVOs about the performance of CAHWs with regard to the treatment function seem to contradict the above statement. This, however, may be due to the fact that only 4 DVOs answered this question on satisfaction.

More than 88% (191/215) of the farmers showed that CAHWs inform them about the name of the disease; 88.8% (191/215) about the cause of the disease; 80.5% (173/215) about transmission of the disease; and 84.2% (181/215) about how the disease can be prevented. Similarly, it was impressive to note that most CAHWs performed clinical examination of animal before treatment, as indicated by 84.7% (182/215) of the farmers interviewed. However, only 49.3% (106/215) of the farmers mentioned that the CAHWs keep treatment books with the farmers and only 38.1% (82/215) of farmers reported that the CAHWs write clinical notes in the books. Treatment books are supposed to be kept with farmers and the CAHWs are expected to write clinical notes whenever they visit and perform treatment.

With regard to record keeping, more than 55% (113/204) of the CAHWs keep treatment records, and 26%(53/204) keep both treatment and production records. More than 40% (82/204), 18% (37/204) and 18% (37/204) of CAHWs interviewed indicated that CAHWs, farmers and DVOs are the users of the records, respectively.

On whether CAHWs conduct follow-up visits, 94.1% (192/204) of farmers agreed that CAHWs carry out follow-up visits to farmers’ kraals following treatment of animals. On such visits, half (50%; 102/204) of the farmers said CAHWs continue treatment of the sick animals, 30.9% (64/204) mentioned that CAHWs provide advice to the farmers, 17.2% (35/204) said they monitor the sick animals and 1.5% (3/204) said CAHWs take samples. When DVOs were asked about the relevance of the CAHWs in these districts, they responded as follows; *“Due to low staffing and high prevalence of zoonotic diseases*, *we give credit to our CAHWs for the tremendous work done towards treatment and from our opinion; CAHWs are relevant in their respective districts*.*”* (Reported by DVO, Napak district.) However, on the aspect of charges for treatment, only 44.7% (96/215) of the farmers were satisfied with the costs charged by the CAHWs.

### Disease surveillance and community mobilization

Results of the performance assessment of the function of surveillance and community mobilization are shown in S3 Table. On the function of disease surveillance, CAHWs were asked to list four notifiable diseases known to them. Thirty three percent (68/204) of them listed Foot and Mouth Disease (FMD) and 26% (53/204) of them listed Contagious Bovine Pleuropneumonia (CBPP) among the notifiable diseases known by the CAHWs. Participation in sample collection, as an aspect of active surveillance, was also assessed, and the results show that 51.5% (105/204) of CAHWs have participated in sample collection, with 38% (79/204) of them having collected fecal samples, 36.3% (74/204) of them having collected tsetse flies and 16.7% (34/204) of them having collected blood samples.

On the use of surveillance reports compiled by CAHWs, the DVOs had this to say;

*“We carry out sensitization*, *field visits*, *treatment*, *vaccination*, *quarantine*, *report to MAAIF but the CAHWs are not catered for in the surveillance budget despite the fact that they carry out surveillance activities”*. As an indication of their involvement in surveillance activities, it was established that CAHWs carry out activities other than treatment of animals during their visits to kraals. In this regard, 21% (42/204) said they collect samples and 59.3% (121/204) said they offer advisory services, among other activities. However, only 19.6% (40/204) and 18.6% (38/204) of respondents said they get some facilitation from NGOs and Central Government, respectively. It was also discovered that the facilitation is irregular and unsustainable, as reported by 41.2% (84/204) of the respondents.

When probed about the type of information included in their surveillance reports, 57.4% (117/204) replied “number of treated animals”, 15.7% (32/204) replied “number of sick animals” and 11.3% (23/204) replied “type of disease identified”. As part of the surveillance function, 50% (102/204) of the CAHWs said they also provide feedback to the farmers on treatment of animals and 38.7% (79/204) said they furnish farmers with upcoming dates of vaccination, if epidemic diseases are identified by this surveillance system. The main methods used to disseminate this information were outreaches (56.4%; 115/204), Kraal visits (14.7%; 30/204) and farmer mobilization (28.9%; 59/204), as reflected by the respective percentages of CAHWs using a particular method.

On the function of community mobilization and sensitization, the CAHWs were not very effective, because only 14% (30/215) of farmers reported their involvement in this activity. Nevertheless, for those who manage to hold community sensitization sessions, 33.5% (72/215) of farmers indicated that they (CAHWs) always relay information of key disease events in the area.

### Control of external parasites

Results of the assessment of their involvement in control of external parasites are shown in S4 Table. On their participation in the control of external parasites 42.2% (86/204) of CAHWs said they participate in spraying of animals. The majority (97.5%; 199/204) of the CAHWs could ably identify the common acaricides used, but only 19.1% (39/204) could correctly tell the classes to which they belong; 52% (106/204) could tell the dilution rates. This clearly depicts the knowledge gap that exists among CAHWs as far as acaricide use and application are concerned. Ticks and tick-borne diseases were reported by 57.2% (123/215) of farmers as a major constraint to livestock production in the region. It was noted that 89% (191/215) of the farmers receive advice on external parasite control from many service providers and that CAHWs contribute substantially in this respect, as noted by 50.2%(108/215) of farmers. Additionally, CAHWs have been found to be instrumental in provision of acaricides, as indicated by 27% (58/215) of the farmers; advising on dilution of acaricides (43.7%; 94/215 farmers) and supervising application of the acaricides (14%; 30/215 farmers).

### Livestock production, reporting and vaccination

Results of the assessment of their involvement in livestock production, reporting and vaccination are shown in S5 Table. With regard to supporting livestock production activities, 96.3% (191/204) of the CAHWs indicated that they also kept livestock and implement improved livestock management practices, like improved feeding (37.7%; 77/204) and breeding (35.8%; 73/204). This finding was also supported by some NGOs, like World Vision working in Abim district. During an interview with the project coordinator, he said, *“they [World Vision] normally encourage farmers to consult CAHWs on animal production and health services because they play a significant role in this respect; however*, *their capacity to support livestock production needs to be strengthened”*.

With regard to assessing disease reporting, it was required that CAHWS mention four diseases that have to be reported. More than 76% (157/204) of the CAHWs listed FMD and 68.6% (140/204) of them listed CBPP among the diseases that must be reported. Whilst all 7 DVOs said that CAHWs were instrumental in reporting animal movements for trade and migrations, only 14.3% (1/7) said CAHWs report animal movements for social cultural practices. However, the DVOs said CAHWs do not report livestock census data; a duty they ought to perform, since they are in touch with kraals, as such data is very useful for planning purposes. With regard to vaccination, the majority of CAHWs (98.5%; 201/204) correctly identified diseases that can be prevented by vaccination. However, only 48% (78/204) could correctly describe how the dosage is determined. More than 95% (195/204) of CAHWs correctly identified the common vaccination sites used. Over 70% (151/215) of the farmers interviewed revealed that the CAHWs normally inform them about contagious diseases in the district. In addition, 59% (127/215) of the farmers reported that CAHWs inform them about laboratory results of samples taken from their farms. More than 77% (167/215) of farmers reported that CAHWs inform them about the advantages of vaccination; and information on their obligations for crush construction, a prerequisite for successful vaccination campaigns, was received by 56.3% (121/215) of the farmers. One of the greatest challenges associated with animal handling is crush construction and this lack has been a major outcry from farmers and CAHWs. **“***We do not have crushes where our large herds of animals can be handled and sprayed well*. *This is one of the biggest problems affecting our community and hindering our work here in Nakapiripirit District*.*”* said a FGD participant in Namalu Sub County, Nakapiripirit District.

### Animal identification and minor surgeries

Assessment of the above functions yielded the following results, detailed in S6 Table. With regard to their participation in minor surgeries, such as dehorning, castration, wound dressing and hoof trimming, 88.7% (181/204) of CAHWs interviewed indicated that the burdizzo was a popular method of castration; while 27% (55/204) said the wire saw was one of the commonly used methods for dehorning. More than 63% (130/204) of the CAHWs said that the reason for dehorning was reduction of injuries whereas 45.1% (92/204) and 67.2% (137/204) of them named reasons for castration as control of breeding and to enhance body weight gain, respectively. Only 11.3% (23/204) of CAHWs could articulate the reasons for hoof trimming. Many of the CAHWs interviewed could not effectively advise farmers on post-operative care, with only 30.4% (62/204) giving advice on feeding, 24.5% (50/204) advising on daily checkup of the animals and 23% (47/204) giving advice on post operative treatment.

More than 82% (169/204) and more than 79% (163/204) of CAHWs felt that branding and ear tags, respectively, were the most popular methods of livestock identification used by farmers in the region. The notices carried on the brands were well understood by the CAHWs, with 64.2% (131/204) reporting that brands carry animal description information, 63.7% (130/204) said they carry ownership data and 57.4% (117/204) reported that brands contain herd location data. However, the general observation was that the benefits of branding were not well appreciated; yet, given the history of conflict and cattle rustling in the area, branding of livestock is vital for proof of ownership and fostering harmony and co-existence.

## Discussion

The delivery of animal health care services in many developing countries remains a major challenge, on account of the low staffing levels, inadequate resources, insecurity and poor infrastructure. Arising from the needs of local communities and from the concerns of local and international agencies, the CAHWs model has been widely adopted in hard-to-reach pastoral areas; albeit taking different approaches [8]. There are numerous examples on the African continent, especially in remote, marginal and conflict-prone pastoral areas, where CAHWs have been instrumental in the delivery of animal health care services, despite their technical and logistical shortcomings. The CAHWs in the Karamoja region of Uganda is one such case. This evaluation exercise has shown that CAHWs are instrumental in treatment of sick animals, disease surveillance and reporting, vaccination, control of external parasites, performing minor surgeries, animal identification, livestock production activities, record keeping and community mobilization. The above facts were highlighted by the findings from the farmers, who indicated that CAHWs are the most readily available animal health care service providers in the region (S2 Table). This is particularly true because of limited veterinary expertise in hard-to-reach areas [8]. However, alone, they are insufficient in meeting all the animal health care needs in marginal pastoral areas; a fact also propounded by Mockshell et al. [4]. The afore mentioned roles played by CAHWs are incontestably important in any livestock production endeavor. However, as our results have shown, the functions have been performed to varying levels, due to the challenges revealed during the focus group discussions (Table 1). For instance, whereas the questionnaire assessment generally revealed that the CAHWs performed well on disease diagnosis and treatment (S2 Table), focus group participants still perceived that many CAHWs face challenges of properly diagnosing and treating key diseases. However, the questionnaire assessment made during the course of this study of how well the CAHWs diagnose and treat diseases was not very rigorous, as we took into consideration their level of training.

It is an undisputed fact that the training offered to the CAHWs is usually of short duration, taking hardly a month. The training normally concentrates around basic livestock health and management issues in the communities [8]. However, since they are only meant to offer primary health care services, the basic knowledge, diagnosis and treatment was considered adequate in assessing performance of this particular function. These observations underscore the need for building more technical capacity and skills in disease management. This is further strengthened by the fact that academic qualifications are not one of the criteria considered in their recruitment. The criteria considered for recruitment include level of activity and residence in an area. The above scenario may partly explain the limited number of CAHWs listing brucellosis as a key local disease (S2 Table and Key informant interview), despite its local importance, according to DVOs. However, the difference in opinion could arise because the disease may not be one of the major causes of production losses in the area, and therefore raises little concern by the CAHWs. The question regarding common diseases in the area was not designed specifically to capture zoonotic diseases, which may explain why CAHWs seemed to under report the disease, despite the DVOs indicating it as a top priority disease, since they have a higher level of awareness of the public health risks.

The lack of formal facilitation from government stems from the unwillingness to integrate the CAWHs into the mainstream civil service. Currently there are challenges in their management and integration in the formal systems [17] and perhaps never will they be formally integrated into the civil service in Uganda. This may be the reason why issues of using old tools and equipment, lack of transport means and other logistical issues were identified as key areas affecting their performance, which affects the sustainability of their services. However, it is also important to note that even the formal government veterinarians face similar challenges. Therefore, for this region, there is a need to train more paraprofessionals who are recognized by the civil service to gradually take over from the CAHWs. These professionals should be deployed strategically at sub-county level and linked to the CAHWs and the drug supply system in the region.

One of the strategic areas targeted for export earnings in Uganda is livestock and livestock products. However, to comply with the Sanitary and Phytosanitary regulations of the World Trade Organization (WTO), developing countries must be able to demonstrate national animal health status by means of scientifically based surveillance efforts [7]. In this regard, CAHWs have been instrumental in disease surveillance of common notifiable diseases in Uganda (S3 Table) because they interact with farmers on a daily basis. Information dissemination is very critical for surveillance and outreach programs are some of the avenues used. Outreaches are community based activities targeting a particular subject matter; for instance, FMD outbreaks. Here, the CAHW goes to the community to address issues relating to a reported FMD outbreak. Community sensitization is another supportive function to surveillance involving awareness creation, and may employ methods like radio talk shows, community posters, community meetings and others. This helps relay important messages to the target communities regarding a particular subject matter. However, because of the various challenges, the CAHWs do not carry out this function appropriately. The other supportive function for surveillance is community mobilization. Community mobilization involves enrolling community members for a given activity, such as a vaccination exercise, or attending a community meeting or other activity. Mobilization entails multiple methods including radio announcements, posters, and telephone calls, among others, and these have also been used to give feedback to farmers.

However, delayed disease reporting, as pointed out in FGDs (Table 1), affects the performance of the surveillance function. In addition, the vast areas of jurisdiction and lack of transport also adversely affects the efficiency of performance of this function. This has adverse effects on disease control. This study has demonstrated that when trained, equipped and supervised, CAHWs can support the identification and reporting of diseases, in addition to collecting biological samples; unlike the situation in Kenya, where Mugunieri et al. [8] observed that they do not collect quantitative data. In fact, in Karamoja the CAHWs have been instrumental in providing ‘hard data’, as Catley and Mariner [7] say, to inform the development of realistic and affordable disease control strategies. The DVOs indicated that CAHWs promptly report disease outbreaks, and actions have always been implemented on the basis of reports made by CAHWs (S3 Table). Actions like quarantine restrictions, vaccinations, treatments, field visits and community sensitizations have been implemented on the basis of reports made by CAHWs. More importantly, their reports have been used to compile monthly epidemiological reports submitted to the Chief Veterinary Officer in the Ministry of Agriculture, Animal Industry and Fisheries. However, the challenges associated with such a task are the lack of diagnostic tools and harsh field conditions. Since these reports eventually end up at the World Organisation for Animal Health (O.I.E.), it is essential that the means of diagnosis are improved and a system of verification of CAHWs’ reports implemented, to ensure accurate reporting. The advent of mobile or portable diagnostic kits and facilities provides an avenue for rapid detection of livestock diseases in such hard-to-reach areas. Nevertheless, due to budgetary constraints in many African countries, this kind of support is unlikely, meaning that the function of surveillance remains largely the role of the public veterinary service providers.

This study also showed that CAHWs were instrumental in tick control activities. However, there were challenges in correctly understanding the classes to which the acaricides belonged and acaricide dilution rates. This clearly depicts the knowledge gap that exists among CAHWs, as far as acaricide use and application are concerned; moreover, the country is currently facing challenges of acaricide resistance that has seen an upsurge in the incidence of tickborne diseases. The CAHWs support the DVOs in sensitizing the farmers on the acaricide types that should be used and how they should be used. In this regard the DVOs advise the CAHWs on the acaricide types to be recommended to the farmers. On the other hand, the CAHWs should provide feedback to the DVOs on the effectiveness of the acaricides. The CAHWs also ought routinely to collect acaricide samples for testing, although this was not mentioned in the evaluation exercise. The DVOs use this information for monitoring acaricide resistance and to notify the Ministry of Agriculture, Animal Industry and Fisheries accordingly. However, the results have shown that there is minimal supervision of acaricide application by the CAHWs; partly because it is not a statutory requirement and also due to a lack of facilitation. This limited involvement may also explain why most CAHWs could not tell the classes to which the acaricides belong, which may hinder selection of appropriate treatments for the locality and exacerbate acaricide resistance development. Overall, from the farmers and CAHWs perspective, there is a need to equip CAHWs with additional knowledge and skills in the management of ticks and tick borne diseases.

The general assessment is that CAHWs have performed well on the function vaccination against diseases like CBPP, CCPP, PPR, Lumpy Skin Disease (LSD), Newcastle disease (NCD) and FMD. The role of CAHWs in vaccination elsewhere in Africa has been well described by Catley [10]. Because of the skeleton veterinary staff, compared to the livestock population, CAHWs permit veterinary services to accomplish these otherwise tough field operations. However, in this study CAHWs were found to be lacking in aspects of dosage determination, cold chain management and vaccine administration.

Available literature has not clearly articulated the role of CAHWs in supporting animal production activities like improved feeding and breeding. Improved livestock management practices form the cornerstone of a more sustainable livestock production. It is well known that in semi-arid climatic conditions, livestock nutrition is a significant challenge that requires intervention strategies for sustainable livestock production. Hence, to attain the goal of poverty eradication and sustainable development in pastoral communities, supporting livestock communities in aspects of animal production is critical, as it ultimately results in improved productivity. The capacity of CAHWs to support livestock production and breeding needs to be strengthened by way of specialized training in aspects of dry season feeding, feed conservation and aspects of breeding. CAHWs in Karamoja have also played a remarkable role have in performing a number of minor surgeries including castration, wound dressing and hoof trimming. However, the evaluation reveals that there is general lack of awareness and understanding of the benefits associated with these minor surgeries by the CAHWs, yet these services are supposed to be paid for by the farmers. This affects the sustainability of offering this service, because if they cannot articulate the benefits of these interventions, farmers are less likely to adopt them. Therefore, there is a need for further training, skills building and retooling the CAHWs for better performance of minor surgeries, as well as promoting their benefits among the local communities.

Being a tribal area, Karamoja region has historically experienced insecurity and hostilities arising from cattle rustling. Some of the measures pursued to stem these challenges are disarmament and livestock identification. Branding and ear tagging are the most popular methods of animal identification and CAHWs played a remarkable role in this respect. Electronic chips are also being used in some circumstances. However, despite being an essential intervention, both farmers and CAHWs seemed not to clearly appreciate and articulate the advantages of animal identification. Indeed, from the focus group discussions conducted in Namalu sub county, Nakapiripirit district, some participants clearly had negative views about the exercise, including *“government seeking to impose taxes on them; government taking away their animals and poisoning their animals in the case of electronic chips*, *that have reportedly killed some animals”*. Therefore strengthening CAHWs and farmers through trainings and organizing outreach programs is vital.

Community mobilization is an important aspect in the performance all functions of CAHWs. Findings indicated that CAHWs have fostered a good level of relationships and trust among the farmers. This CAHWs approach has empowered farmers to participate in decision-making processes regarding their farming enterprises and the type of animal health care services they receive. Similar observations were made by Mugunieri et al. [8] about the delivery of animal health care services by community-based animal healthcare services in Kenya. Therefore strengthening CAHWs in community mobilization and engagement skills and strategies is paramount.

## Conclusions and recommendations

CAHWs have played a vital role in providing primary animal healthcare services, which are a cornerstone for improving and sustaining livestock productivity in the Karamoja region. This study found that with continuous training, logistical support and close supervision by the public sector veterinarians, CAHWs could contribute enormously to the provision of primary animal health care services where the traditional extension system is inadequate. Continuous training in disease identification and reporting, disease surveillance using participatory approaches, minor surgeries, dry season feeding, records keeping and animal identification are essential for more effective animal health care service delivery. Community sensitization on the different methods of animal identification and their benefits needs to be conducted. For sustainability, continuous training and strategic deployment of paraprofessionals that are formally recognised by the traditional civil service is recommended as an approach to sustain the delivery of primary animal health care services in Karamoja.

## Supporting information

S1 Table Criteria grid. (DOCX)

S2 Table CAHWs involvement in treatment, record keeping and their technical abilities. (DOCX)

S3 Table Notifiable diseases, disease surveillance and community mobilization. (DOCX)

S4 Table CAHW involvement in the control of external parasites. (DOCX)

S5 Table Livestock production, reporting and vaccination. (DOCX)

S6 Table CAHWs involvement in minor surgeries and animal identification. (DOCX)

S1 Text Interview guide for NGO’s/Key informants. (DOCX)

S2 Text Farmers questionnaire. (DOCX)

S3 Text CAHWs questionnaire. (DOCX)

S4 Text DVO’s questionnaire. (DOCX)
