# Supplementary material for: Participatory evaluation of delivery of animal health care services by community animal health workers in Karamoja region of Uganda
Source: PLoS One. 2017 Jun 8;12(6):e0179110. doi: 10.1371/journal.pone.0179110 (PMC5464622; doi:10.1371/journal.pone.0179110)
Supplement: S1 Table — (DOCX) [file pone.0179110.s001.docx]

**Table 2: Criteria grid**

| **Function** | **Criteria suggested by focus groups** | **Revised criteria** | **Stakeholders** | **Questions** |
| --- | --- | --- | --- | --- |
| Treatment | - Report from the farmers - Taking of history - Physical examination of the sick animal - Tentative diagnosis - Drug prescription and administration - Record keeping - General advice to the farmer - Follow up | - Availability to farmers - Type of information provided to farmers - Technical ability - Follow up visits - Types of records kept - Cost of treatment | Farmers  Farmers  DVO/VO/AHO  Farmers  Farmers  CAHWs  CAHWs  DVO  NGO | **Availability to farmers**   - Do you have CAHWs in your area? - Do they visit you when you request? - How long does it take them to visit you when you call?   **Type of information provided to farmers**   - Do they tell you the name of the disease they treat? - Do they tell you the cause of the disease? - Do they tell you how the disease is transmitted? - To they give you information on prevention?   **Technical ability**   - Do they examine the animals before treatment? - What examination do they do?   **Follow up visits**   - Do they carry out follow up visits after treatment?   **Types of records kept**   - Do you have a treatment book? - Do CAHWs write any clinical notes in a treatment book   **Cost of treatment**   - Is the cost of treatment affordable in your opinion? |
|  |  |  |  | **Technical ability**   - Identify common diseases in the locality - Ability to prescribe appropriate treatment   **Types of records kept**   - Mention the types of records kept - Mention the uses of these records - Who uses these records?   **Follow up visits**   - Do you do follow up visits? - What do you do during those visits?   **Type of information provided to farmers**   - Mention the type of information given to farmers during your visits |
|  |  |  |  | **Technical ability**   - What criteria did you use to select CAHWs? - What training have they acquired? - What is your opinion on their performance regarding treatment? - How often do you hold support supervision meetings/ field visits? - What is your opinion on their relevance in the district |
|  |  |  |  | **Availability to farmers**   - Do you work with CAHWs - What activities are they involved in? |
| Disease Surveillance | - Mobilization and sensitization of the communities - Making monthly visits to farmers - Identify common diseases and inform the DVO office - Frequent and timely reporting - Monitoring disease situation especially those identified and those emerging | - Participation in community mobilization and sensitization - Periodic visits to Kraals/farms - Surveillance reports - Ability to identify common diseases - Support to surveillance | Farmers  Farmers  Farmers  CAHWs  CAHWs  DVOs, AHOs/VOs  DVOs/NGOs  DVO/NGO  DVOs/NGO  DVO/NGO | **Participation in community mobilization and sensitization**   - Do CAHWs hold farmers meetings? - List type of information delivered in the meetings   **Periodic visits to Kraals/farms**   - Do CAHWs Visit Kraals when not invited - Mention activities done by CAHWs on such visits   **Surveillance reports**   - To whom do you report disease incidences in your animals? - Do CAHWs inform you of disease outbreaks in other areas? |
|  |  |  |  | **Ability to identify common diseases**   - List the diseases commonly reported in your area? - List 4 notifeable diseases you know   **Periodic visits to Kraals/farms**   - List other reasons for visiting kraals other than treatment - Who facilitates these visits? - How often do you receive this facilitation?   **Surveillance Reports**   - Identify the data type you include in the report - List the type of feedback given to farmers? - Identify the methods you use to give feedback to farmers |
|  |  |  |  | **Surveillance Reports**   - How long do they take to report outbreaks? - What form of reports? (Verbal, written, electronic, call?) - Level of satisfaction with reports - Is the data provided reflected in the monthly Epidemiological report of MAAIF - List interventions you have ever implemented on the basis of these reports   **Support to surveillance**   - Are CAHWs catered for in the budget for disease surveillance? - Other than financial support name the other form of support you have extended to the CAHWs. |
|  |  |  |  | **Support to surveillance**   - Do you extend any support to CAHWs towards disease surveillance? - Mention the type of support - List the activities you have supported in the past in regard to surveillance   **Surveillance Reports**   - Do you receive copies of surveillance reports? - Who provides the reports? - What is your opinion on the quality of the reports? - List the interventions you have ever implemented on the basis of the surveillance reports |
| 3. Control of external and internal parasites | - Community mobilization and sensitization - Routine spraying and de-warming - Demonstrations through kraal out reaches | - Involvement in community mobilization and sensitization for external parasite control - Involvement in Routine spraying of animals - Demonstrations through kraal out reaches - Technical ability | Farmer/Kraal leader  CAHWs  CAHWs  CAHWs | **Involvement in community mobilization and sensitization for external parasite control**   - Which are the common parasites affecting your animals - Do you receive any advise on their control? - Who gives this advice? - How is this advice given to farmers? - How often do you receive this advice?   **Involvement in Routine spraying of animals**   - How do you control external parasites? - What role do CAHWs play in this exercise? - How often do they get involved? |
|  |  |  |  | **Involvement in Routine spraying of animals**   - List the activities you carry out related to spraying of animals - Are there official facilities where you organize these activities - Name these facilities (e.g. communal crushes, spray races, dips) - How are these activities facilitated? - Are the drugs readily available   **Demonstrations through kraal out reaches**   - How many outreaches can you perform in a month? - List the items required for these outreach demonstrations? - Who provides these items? - What exactly do you do during these outreaches?   **Technical ability**   - Ability to identify common acaricides and their classes - Ability to mention mode of application - Ability to tell the dilution rate |
| 4. support to Livestock production | Advice farmers on animals health, feeding, housing and routine management | - Ability to demonstrate the best modes of production - Ability to advise farmers on animal production | Farmers/kraal leaders  CAHWs | **Ability to demonstrate the best modes of production**   - Is your CAHW involved in livestock production? - Do you visiting the CAHW and learn from their kraal/farms?   **Ability to advise farmers on animal production**   - Do CAHWs give you advice to farmers about production and economic analysis? - List the areas where you have received advise (e.g. feeding, breeding etc) |
|  |  |  |  | **Ability to advise farmers on animal production**   - Whether CAHW keeps animals and what type - Whether CAHW implements improved techniques in his/her farm and in what fields (i.e. breeding, feeding, housing etc.) |
| 5. Reporting | Get information from farmers  Identify the diseases and make a report  Know the type of record to keep  Count the number and record the item  Know the duration in which your carry out the record  Know the specific time  Keep records | - Advice to farmers about diseases - CAHWs involvement in reporting - Knowledge about official diseases to report | Farmers  DVO  CAHWs | **Advice to farmers about diseases**   - Do CAHWs inform you about contagious disease in the district or about the analysis results after samples were taken from your farm? - When you particularly fear for your animal’s health whom do you call? |
|  |  |  |  | **CAHWs involvement in reporting**   - Is CAHW regularly invited to meetings? - Does the CAHW follow DVOs recommendations after outbreak? - Does CAHW report high morbidity/mortality in less than 12hrs? - Does the CAHW report to DVO animal movements and census? |
|  |  |  |  | **Knowledge about official diseases to report**   - What are the four diseases that you have to report? |
| Vaccination | Mobilization of farmers  Sensitization  Training of CAHWs  Organize cattle kraal  Vaccinate  Reporting | - CAHWs involvement in official vaccination campaigns - CAHWs involvement in official vaccination campaigns - The setting up of campaigns outside official ones - The CAHWs technical abilities | Farmers /kraal leaders  DVO  CAHWs | **CAHWs involvement in official vaccination campaigns**   - Does the CAHW inform you about the benefits and advantages of vaccination? - Does the CAHW give advice on the care of animals post vaccination? |
|  |  |  |  | **CAHWs involvement in official vaccination campaigns**   - Is there a place where CAHWs use to organize vaccination campaigns? - Did you receive a report of each vaccination campaign?   **The setting up of campaigns outside official ones**   - Does the CAHW carry out vaccination in the framework of government or NGO campaign |
|  |  |  |  | **The CAHWs technical abilities**   - How do you store your vaccines? - List 5 requirements for a successful vaccination exercise |
| 7. Minor surgery (De-horning, castration and hoof trimming) | - Need a rope to restrain the animals - Need a de-horning wire - Need a razor blade/knife - Need a hot iron - Need drugs e.g. pen strep, cotton wool, healing oil - Restrain the animals, cut the skin at the base of the horn, place the wire at the cut area of the horn and cut with the wire, place the hot iron on the cut area, close the hole with cotton wool, spray with supona and inject with penstrep | - Involvement in Dehorning and Castration - Technical ability | Farmers  CAHWs | **Involvement in Dehorning and Castration**   - Do you de-horn or castrate your animals? - If there is a need for dehorning or castration whom do you call? - Does the CAHW give you advise on post castration/dehorning care? - Is the cost affordable? |
|  |  |  |  | **Technical ability**   - List the some of the requirements you need for dehorning and castration - List 2 reasons for dehorning - List 3 reasons for castration - Mention the type of advise you give to farmers post castration dehorning |
| Animal Identification/branding |  | - Involvement in animal identification/branding - Technical ability | Farmers  CAHWs  DVO, VO | **Involvement in animal identification/branding**   - Do you brand /identify your animals? - Is the branding/identification a private or an official arrangement? - Who does the branding/identification? |
|  |  |  |  | **Technical ability**   - List 3 types of animal identification you normally use - What information do the brands/other identification carry? - List 3 uses of branding / identification |
|  |  |  |  | **Involvement in animal identification/branding**   - Are CAHWs involved in branding/Identification of animals? - Is the branding/identification a private or an official arrangement? - Who facilitates these exercises - Do CAHWs provide reports concerning these exercises? |
| 9.Sustainability |  | - Support given to the CAHW | DVO | **Support given to the CAHW**  What kind of support do you provide to CAHWs  Are CAHWs Activities provided for in the Annual budgets? |
