# Supplementary material for: Participatory evaluation of delivery of animal health care services by community animal health workers in Karamoja region of Uganda
Source: PLoS One. 2017 Jun 8;12(6):e0179110. doi: 10.1371/journal.pone.0179110 (PMC5464622; doi:10.1371/journal.pone.0179110)
Supplement: S2 Table — (DOCX) [file pone.0179110.s002.docx]

Table 1: CAHWs involvement in treatment, record keeping and their technical abilities (n= Farmers (215), CAHWs (204), DVO’s (7))

| **Variable** | **Respondent** | **Category** | **Frequency** | **Percentage (%)** |
| --- | --- | --- | --- | --- |
| Common Diseases in the Region | CAHWs | Anaplasmosis  CBPP  ECF  CCPP  FMD  PPR  Mange  Brucellosis  Babesiosis  Lumpy Skin Disease  Helminthes  Pneumonia | 154  144  109  72  43  42  24  22  20  13  9  1 | 75.5  70.6  53.4  35.3  21.1  20.6  11.8  10.8  9.8  6.4  4.4  0.5 |
| Main Clinical Signs of Disease 1  Main Clinical Signs of Disease 2  Main Clinical signs of disease 3 | CAHWs | Correct  Incorrect  Correct  Incorrect  Correct  Incorrect | 186  18  167  37  170  34 | 91.2  8.8  81.9  18.1  83.3  16.7 |
| Treatment of Disease 1  Treatment of Disease 2  Treatment of disease 3 | CAHWs | Correct  Incorrect  Correct  Incorrect  Correct  Incorrect | 173  31  161  43  159  45 | 84.8  15.2  78.9  21.1  77.9  22.1 |
| Main Animal Health Care Service Providers | Farmers | Government veterinarians  Private veterinarians  CAHWs  Drug dealers  NGOs, CBOs  Traditional healers | 77  32  171  26  45  16 | 35.8  14.9  79.5  12.1  20.9  7.4 |
| Most available when Needed | Farmers | Government veterinarians  Private veterinarians  CAHWs  Drug dealers  NGOs, CBOs  Traditional healers | 35  14  150  5  8  3 | 16.3  6.5  69.8  2.3  3.7  1.4 |
| Do CAHWs visit you when you request? | Farmers | All the time  Most of the time  Some times  Rarely  Never | 63  52  59  32  09 | 29.3  24.2  27.4  14.9  4.2 |
| How long does it take CAHWs to visit you when you call? | Farmers | 12hours  24 hours  2-3 hours  1 week  Over 1 week | 122  23  27  17  26 | 56.7  10.7  12.6  7.9  12.1 |
| Do the CAHWs tell you the name of the disease they treat? | Farmers | Yes  No | 191  24 | 88.8  11.2 |
| Do the CAHWs tell you the cause of the disease? | Farmers | Yes  No | 176  39 | 88.8  11.2 |
| Do the CAHWs tell you how the disease is transmitted? | Farmers | Yes  No | 173  42 | 80.5  19.5 |
| Do the CAHWs give you information on prevention? | Farmers | Yes  No | 181  34 | 84.2  15.8 |
| Do the CAHWs examine the animals before treatment? | Farmers | Yes  No | 182  33 | 84.7  15.3 |
| Do the CAHWs carry out follow up visits after treatment? | Farmers | Yes  No | 156  59 | 72.6  27.4 |
| Do CAHWs have treatment books? | Farmers | Yes  No | 106  109 | 49.3  50.7 |
| Do they write any clinical notes in the treatment book? | Farmers | Yes  No | 82  133 | 38.1  61.9 |
| Type of Records Kept | CAHWs | Treatment Records  Production Records  Both  Don’t Know | 113  10  53  28 | 55.4  4.9  26.0  13.7 |
| Who uses the Records | CAHWs | CAHWs  Farmers  DVO’s  Ministry  Don’t know | 82  37  37  17  31 | 40.2  18.1  18.1  8.3  15.2 |
| Satisfaction with cost of treatment | Farmers | Very satisfied  Satisfied  Somehow satisfied  Not satisfied | 25  96  59  35 | 11.6  44.7  27.4  16.3 |
| Criteria for selecting CAHWs | DVOs | Academic qualification  Residence in area  Level of activity  Livestock ownership  Others | 00  03  04  00  00 | 0.0  42.9  57.1  0.0  0.0 |
| Training acquired | DVOs | Disease surveillance and reporting  Disease diagnosis and treatment  Records keeping  Vaccine handling and vaccination  Meat inspection and Public health  Animal Husbandry practices | 3  4  3  2  2  4 | 42.9  57.1  42.9  28.6  28.6  57.1 |
| Satisfaction with performance of the treatment function | DVOs | Good  Poor | 3  1 | 42.8  14.2 |
| Opinion on relevance of CAHWs | DVOs | Very Relevant  Relevant | 2  5 | 28.6  71.4 |
