# Supplementary material for: Participatory evaluation of delivery of animal health care services by community animal health workers in Karamoja region of Uganda
Source: PLoS One. 2017 Jun 8;12(6):e0179110. doi: 10.1371/journal.pone.0179110 (PMC5464622; doi:10.1371/journal.pone.0179110)
Supplement: S3 Table — (DOCX) [file pone.0179110.s003.docx]

Table 5: Notifiable diseases, disease surveillance and community mobilization (n= Farmers (215), CAHWs (204), DVO’s (7))

| **Variable** | **Respondent** | **Category** | **Frequency** | **Percentage** |
| --- | --- | --- | --- | --- |
| Notifiable diseases Known to CAHWs | CAHWs | FMD  CBPP  PPR  CCPP  ECF  Anaplasmosis  Brucellosis | 68  53  29  22  12  12  8 | 33.0  26.0  14.0  11.0  6.0  6.0  4.0 |
| Samples Collected | CAHWs | Fecal  Blood  Tsetse flies  Pus | 79  34  74  17 | 38.7  16.7  36.3  8.3 |
| Reasons for Visiting Kraals other than Treatment | CAHWs | Sample collection  Advisory services  Minor surgical operations  Assess rate of animal production  Don’t know | 42  121  32  2  7 | 20.6  59.3  15.7  1.0  3.4 |
| Who facilitates these field visits | CAHWs | Farmers  Central/ local government  NGOs  Self  Do not know | 39  38  40  82  5 | 19.1  18.6  19.6  40.2  2.5 |
| How often do you receive this facilitation? | CAHWs | Weekly  Monthly  Every 2 months  Quarterly  Not regularly  Never | 47  36  6  20  84  11 | 23.0  17.6  2.9  9.8  41.2  5.4 |
| Identify the type of information you include in the report | CAHWs | Number of animal treated  Number of animals sick  Number of animals dead  Location  Number Survived  Type of disease  Disease infrastructure | 117  32  16  10  2  23  2 | 57.4  15.7  7.8  4.9  1.0  11.3  1.0 |
| The type of feedback given to farmers | CAHWs | Treating animals  Dates of vaccination  Monitoring | 102  79  23 | 50.0  38.7  11.3 |
| Methods used to give feedback to farmers | CAHWs | Mobilization  Outreaches  Kraal visits | 59  115  30 | 28.9  56.4  14.7 |
| Do CAHWs hold farmers meetings? | Farmers | Often times  Some times  Rarely  Never | 30  69  53  63 | 14.0  32.1  24.7  29.3 |
| Mention the type of information delivered in the meetings | Farmers | Key disease events in the area  Disease events in neighboring areas  Planned activities  Reports of finished activities  Animal Movements | 72  56  26  9  11 | 33.5  26.0  12.1  4.2  5.1 |
| Do CAHWs visit kraals when not invited | Farmer | Often times  Some times  Rarely  Never | 32  66  57  60 | 14.9  30.7  26.5  27.9 |
| Mention activities done by CAHWs on such visits | Farmer | Gather information on diseases  Collect samples  Give advice on disease control  Deliver news of disease events in area  Deliver reports of activities | 99  26  114  66  18 | 55.6  14.6  64.0  37.1  10.1 |
| To whom do you report disease incidences in your animals? 152.6% (n=328) | Farmer | Government veterinarians  Private veterinarians  CAHWs  Drug dealers  NGOs, CBOs  Traditional healers | 89  19  160  24  31  5 | 41.4  8.8  74.4  11.2  14.4  2.3 |
| Do CAHWs inform you of disease outbreaks in other areas? | Farmers | Often times  Some times  Rarely  Never | 66  81  45  23 | 30.7  37.7  20.9  10.7 |
| Do CAHWs report Disease Outbreaks | DVOs | Yes  No | 7  00 | 100.0  0.0 |
| How long do CAHWs take to report Outbreaks | DVOs | 12 – 24 hours  2 -7 days | 4  3 | 57.1  42.9 |
| Satisfaction with the reports | DVOs | Satisfied  Somehow satisfied | 4  3 | 57.1  42.9 |
| Is data reflected in MAAIF epidemiology reports | DVOs | Yes  No | 6  1 | 85.7  14.3 |
| Interventions carried out on the basis of these reports | DVOs | Sensitization  Field visits  Treatment  Vaccination  Quarantine restrictions  Submit reports to MAAIF | 3  6  2  7  5  3 | 42.9  85.7  28.6  100.0  71.4  42.9 |
| Support to surveillance | DVOs | Refresher training  Linkage to Drug companies and other development partners  Provision of PPE | 6  7  5 | 85.7  100.0  71.4 |
