# Supplementary material for: Participatory evaluation of delivery of animal health care services by community animal health workers in Karamoja region of Uganda
Source: PLoS One. 2017 Jun 8;12(6):e0179110. doi: 10.1371/journal.pone.0179110 (PMC5464622; doi:10.1371/journal.pone.0179110)
Supplement: S4 Table — (DOCX) [file pone.0179110.s004.docx]

Table 6: CAHW involvement in the control of external parasites (n= Farmers (215), CAHWs (204), DVO’s (7))

| **Variable** | **Respondent** | **Category** | **Frequency** | **Percentage %** |
| --- | --- | --- | --- | --- |
| Activities carried out related to spraying of animals | CAHWs | Restraining animals  Spraying animals  Mobilization | 55  86  63 | 27  42.2  30.9 |
| Are there official facilities where you organize these activities | CAHWs | Yes  No | 129  75 | 63.2  36.8 |
| Facilities naming | CAHWs | Communal crushes  Holding grounds  Spray races  Dips | 103  66  20  6 | 50.5  32.4  9.8  2.9 |
| Are the acaricides readily available? | CAHWs | Yes  No | 144  60 | 70.6  29.4 |
| How many out reaches can you perform in a month | CAHWs | Once  Twice  Thrice | 53  69  82 | 26.0  33.8  40.2 |
| Items required for these outreach demonstrations | CAHWs | Transport  Pen  Note book  Drugs  Personal Protective gear | 44  7  15  102  36 | 21.6  3.4  7.4  50.0  17.6 |
| Who provides these items? | CAHWs | Farmers  Central government  NGOs  CBOs  Self-facilitation (savings) | 17  35  105  7  40 | 8.3  17.2  51.5  3.4  19.6 |
| What exactly do you do during these outreaches? | CAHWs | Advising farmers  Mobilization  Spraying /treatment | 75  27  102 | 36.8  13.2  50.0 |
| Knowledge on Common acaricides that are used in tick/ fly control | CAHWs | Correct  Incorrect | 199  5 | 97.5  2.5 |
| What are their classes | CAHWs | Correct  Incorrect | 39  165 | 19.1  80.9 |
| What are the main modes of application? | CAHWs | Topical  Spray  Dip | 43  149  12 | 21.1  73.0  5.9 |
| How do you dilute the acaricides above? | CAHWs | Correct  Incorrect | 106  98 | 52.0  48.0 |
| Which are the common parasites affecting your animals? | Farmer | Ticks  Mites  Worms  Flies  *Other | 123  24  32  34  2 | 57.2  11.2  14.9  15.8  0.9 |
| Do you receive any advice on their control? | Farmer | Yes  No | 191  24 | 88.8  11.4 |
| Who gives this advice? | Farmer | Government veterinarians  Private veterinarians  CAHWs  Drug dealers  NGOs, CBOs  Traditional healers | 70  25  108  7  4  1 | 32.6  11.6  50.2  3.3  1.9  0.5 |
| How is this advice given to farmers? | Farmer | Community meetings  Community outreaches  During visit by farmer to drug shops  During casual interactions with CAHWs | 84  58  23  22 | 39.1  27.0  10.7  10.2 |
| How often do you receive this advice? | Farmer | Often times  Some times  Rarely  Never | 44  109  54  8 | 20.5  50.7  25.1  3.7 |
| How do you control external parasites? | Farmer | Hand picking  Spraying  Dipping  Pour on | 73  129  8  2 | 34.0  60.0  3.7  0.9 |
| What role do CAHWs play in this exercise? | Farmer | Provide Acaricides  Advise on dilution  Supervise application of Acaricides  Collecting information on activity  Sensitization on external parasite control | 58  94  30  6  16 | 27.0  43.7  14.0  2.8  7.4 |
