# Supplementary material for: Participatory evaluation of delivery of animal health care services by community animal health workers in Karamoja region of Uganda
Source: PLoS One. 2017 Jun 8;12(6):e0179110. doi: 10.1371/journal.pone.0179110 (PMC5464622; doi:10.1371/journal.pone.0179110)
Supplement: S5 Table — (DOCX) [file pone.0179110.s005.docx]

Table 7:Livestock production, reporting and vaccination (n= Farmers (215), CAHWs (204), DVO’s (7))

| **Variable** | **Respondent** | **Category** | **Frequency** | **Percentage %** |
| --- | --- | --- | --- | --- |
| Do you keep animals? | CAHWs | Yes  No | 191  13 | 93.6  6.4 |
| What kinds of animals do you keep? | CAHWs | Cattle  Goats  Chicken  Sheep  Donkeys  Ducks | 159  169  133  141  78  90 | 77.9  82.8  65.2  69.1  38.2  44.1 |
| What livestock management practices do you implement? | CAHWs | Breeding  Feeding  Housing  None | 73  77  53  1 | 35.8  37.7  26.0  0.5 |
| What are the diseases that you have to report? | CAHWs | FMD  PPR  CBPP  LSD | 157  76  140  54 | 77.0  37.3  68.6  26.5 |
| Diseases that can be prevented by vaccination | CAHWs | Correct  Incorrect | 201  3 | 98.5  1.5 |
| Vaccine storage and management technique | CAHWs | Fridge  Cool boxes  Ice packs  Tree shades | 70  89  34  11 | 34.3  43.6  16.7  5.4 |
| Requirements for a successful vaccination exercise | CAHWs | Overall  Face mask  Gloves  Gumboots  Vaccine | 43  45  50  49  152 | 21.1  22.1  24.5  24.0  74.5 |
| How do you determine the dosage for vaccination? | CAHWs | Correct  Incorrect | 78  106 | 48.0  52.0 |
| Vaccination sites for common vaccines used. | CAHWs | Correct  Incorrect | 195  9 | 95.6  4.4 |
| Does your CAHW own any animals? | Farmers | Yes  No  Not aware | 177  16  22 | 82.3  7.4  10.2 |
| What kind of bulls do you keep for mating? | Farmers | Strong  Active  Healthy  Fast growing  Other | 66  89  40  18  02 | 30.7  41.4  18.6  8.4  0.9 |
| Does the CAHW assist you to select the right bulls for mating? | Farmers | Yes  No | 78  137 | 36.3  63.7 |
| Do you visit the CAHW and learn from their kraal/ farms? | Farmers | Often times  Some times  Rarely  Never | 22  80  45  68 | 10.2  37.2  20.9  31.6 |
| Do CAHWs give you advice about animal production? | Farmers | Often times  Some times  Rarely  Never | 30  88  42  55 | 14.0  40.9  19.5  25.6 |
| Besides natural grazing what else do your animals feed on? 169.8% (n=365) | Farmers | Acacia pods  Mineral licks  Crop residue  Brewers waste  Domestic refuse | 65  74  91  89  46 | 30.2  34.4  42.3  41.4  21.4 |
| Does your CAHW advise you on alternative feeding? | Farmers | Yes  No | 105  110 | 48.8  51.2 |
| Do CAHWs inform you about contagious disease in the district? | Farmers | Often times  Some times  Rarely  Never | 72  79  32  32 | 33.5  36.7  14.9  14.9 |
| Do CAHWs inform you about the analysis results after samples were taken from your farm? | Farmers | Often times  Some times  Rarely  Never | 34  54  36  91 | 15.8  25.1  16.7  42.3 |
| When faced with livestock health challenges whom do you call? 148.4% (n=319) | Farmers | Government veterinarians  Private veterinarians  CAHWs  Drug dealers  NGOs, CBOs  Traditional healers | 89  23  154  20  26  7 | 41.4  10.7  71.6  9.3  121  3.3 |
| Does the CAHW inform you about the benefits and advantages of vaccination? | Farmers | Often times  Some times  Rarely  Never | 93  74  27  21 | 43.3  34.4  12.6  9.8 |
| Does the CAHW give advice on the care of animals post vaccination? | Farmers | Often times  Some times  Rarely  Never | 56  76  45  38 | 26.0  35.3  20.9  17.7 |
| Which of the following responsibilities regarding vaccination does the CAHW inform you about? 147.4% (n= 317) | Farmers | Cost sharing  Selection of the right age for vaccination  Crush construction  Contra indications for vaccination | 85  70  121  41 | 39.5  32.6  56.3  19.1 |
| Are CAHWs regularly invited to meetings? | DVOs | Yes  No | 6  1 | 85.7  14.3 |
| Do the CAHWs follow DVOs’ recommendations after outbreak? | DVOs | All the time  Most of the time  Some times  Rarely | 2  1  3  1 | 28.6  14.3  42.9  14.3 |
| Do you receive reports from CAHWs on the following? | DVOs | Animal movement for trade  Animal movement for migrations  Animal movement for social cultural practices  Census | 7  7  1  0 | 100.0  100.0  14.3  0.0 |
| CAHWs’ participation in selection of vaccination sites | DVOs | Yes  No | 7  0 | 100.0  0.0 |
| CAHWs participate in vaccination against the following disease | DVOs | PPR  NCD  FMD  LSD  Brucellosis | 7  7  7  3  1 | 100.0  100.0  100.0  42.9  14.3 |
