# Supplementary material for: Participatory evaluation of delivery of animal health care services by community animal health workers in Karamoja region of Uganda
Source: PLoS One. 2017 Jun 8;12(6):e0179110. doi: 10.1371/journal.pone.0179110 (PMC5464622; doi:10.1371/journal.pone.0179110)
Supplement: S6 Table — (DOCX) [file pone.0179110.s006.docx]

Table 8: CAHWs involvement in minor surgeries and animal identification (n= Farmers (215), CAHWs (204), DVO’s (7))

| **Variable** | **Respondent** | **Category** | **Frequency (n)** | **Percentage %** |
| --- | --- | --- | --- | --- |
| Requirements for dehorning | CAHWs | Wire saw  Crush  Rope | 55  12  53 | 27.0  5.9  26.0 |
| Requirements for castration | CAHWs | Burdizzo  Crush  Rope | 181  8  84 | 88.7  3.9  41.2 |
| Dehorning reduces injuries | CAHWs | Yes  No | 130  74 | 63.7  36.3 |
| Is the wire saw a method used for dehorning? | CAHWs | Yes  No | 15  189 | 7.4  92.6 |
| Methods of castration | CAHWs | Correct  Incorrect | 102  102 | 50.0  50.0 |
| Reasons for castration | CAHWs | Body weight gain  Control breeding | 137  92 | 67.2  45.1 |
| Reasons for hoof trimming | CAHWs | Foot rot control  Disease control | 23  17 | 11.3  8.3 |
| Type of advice given to farmers post castration and dehorning | CAHWs | Good feeding  Restraining animals  Daily checkup of animals  Treatment | 62  45  50  47 | 30.4  22.1  24.5  23.0 |
| Problems encountered during a minor surgery | CAHWs | Bleeding  Death  Both Bleeding and death  Restraining animals  None | 91  39  8  6  60 | 44.6  19.1  3.9  2.9  29.4 |
| Types of animal identification used | CAHWs | Color  Brand  Ear tags  Ear notch | 22  169  163  136 | 10.8  82.8  79.9  66.7 |
| Content of animal IDs | CAHWs | Animal description  Ownership data  Location data | 131  130  117 | 64.2  63.7  57.4 |
| Uses of animal identification | CAHWs | Easy identification  Memory reference | 174  2 | 85.3  1.0 |
| Do you de-horn or castrate your animals? | Farmers | Yes  No | 199  16 | 92.0  7.4 |
| If there is a need for dehorning or castration whom do you call? | Farmers | Government veterinarians  Private veterinarians  CAHWs  Drug dealers  NGOs, CBOs  Traditional healers | 28  34  99  03  29  22 | 13.0  15.8  46.0  1.4  13.5  10.2 |
| Do CAHWs give you advice on post castration/ dehorning care? | Farmers | Often times  Some times  Rarely  Never | 48  75  43  49 | 22.3  34.9  20.0  22.8 |
| Do they charge you for castration and dehorning? | Farmers | Yes  No | 99  116 | 46.0  54.0 |
| Are you satisfied with the cost of dehorning or castration? | Farmers | Very satisfied  Satisfied  Somehow satisfied  Not satisfied | 71  71  30  43 | 33.0  33.0  14.0  20.0 |
| Do you brand/ identify your animals? | Farmers | Yes  No | 196  19 | 91.2  8.8 |
| Is the branding/identification a private or an official arrangement? | Farmers | Official  Private  Both | 81  129  5 | 37.7  60.0  2.3 |
| Who does the branding/ identification? | Farmers | Government veterinarians  Private veterinarians  CAHWs  Drug dealers  NGOs, CBOs  Traditional healers | 65  28  60  8  22  32 | 30.2  13.0  27.9  3.7  10.2  14.9 |
| CAHWs involvement in animal identification/ branding | DVOs | Yes  No | 5  2 | 71.4  28.6 |
| Who sponsors animal branding exercises | DVOs | OPM | 7 | 100.0 |
| How often do CAHWs provide reports about branding exercise | DVOs | Occasionally  Never | 3  4 | 42.9  57.1 |
