# Supplementary material for: Participatory evaluation of delivery of animal health care services by community animal health workers in Karamoja region of Uganda
Source: PLoS One. 2017 Jun 8;12(6):e0179110. doi: 10.1371/journal.pone.0179110 (PMC5464622; doi:10.1371/journal.pone.0179110)
Supplement: S1 Text — (DOCX) [file pone.0179110.s007.docx]

**EVALUATION OF PERFORMANCE OF CAHWS IN KARAMOJA REGION - UGANDA**

**INTERVIEW GUIDE FOR NGO’S / KEY INFORMANTS**

**DISTRICT……………………………………NGO NAME…………………………**

**DATE ………………………………**

1. Do you work with CAHWs
2. What activities are they involved in?
3. Do you extend any support to CAHWs towards disease surveillance?
4. Mention the type of support
5. List the activities you have supported in the past in regard to surveillance
6. Do you receive copies of surveillance reports?
7. Who provides the reports?
8. What is your opinion on the quality of the reports?
9. List the interventions you have ever implemented on the basis of the surveillance reports
