# Supplementary material for: Participatory evaluation of delivery of animal health care services by community animal health workers in Karamoja region of Uganda
Source: PLoS One. 2017 Jun 8;12(6):e0179110. doi: 10.1371/journal.pone.0179110 (PMC5464622; doi:10.1371/journal.pone.0179110)
Supplement: S2 Text — (DOCX) [file pone.0179110.s008.docx]

QUESTIONNAIRE NUMBER……………………………………………………….

**EVALUATION OF PERFORMANCE OF CAHWS IN KARAMOJA REGION - UGANDA**

**FARMERS QUESTIONNAIRE**

DATE OF INTERVIEW………………………………………………………………

DISTRICT……………………………………SUBCOUNTY………………………

VILLAGE (LC1)………………………………………………………………………

TELEPHONE CONTACT OF RESPONDENT……………………………………..

NAME OF INTERVIEWER………………………………………………………….

TELEPHONE CONTACT OF INTERVIEWER**……………………………………**

**SECTION A: TREATMENT**

**A1: Availability to Farmers**

1. Who are the animal health service providers
2. Government veterinarians
3. Private vets
4. CAHWs
5. Drug dealers
6. NGOs, CBOs
7. Traditional Healers
8. Who are the most available?
9. Government veterinarians
10. Private vets
11. CAHWs
12. Drug dealers
13. NGOs, CBOs
14. Traditional Healers
15. Do CAHWs visit you when you request?
16. All the time
17. Most of the time
18. Some times
19. Rarely
20. Never
21. How long does it take CAHWs to visit you when you call?
22. 12 hours
23. 24 hours
24. 2 – 3 days
25. 1 week
26. Over 1 week

**Type of information provided to farmers**

1. Do the CAHWs tell you the name of the disease they treat?
2. Yes
3. No
4. Do the CAHWs tell you the cause of the disease?
5. Yes
6. No
7. Do the CAHWs tell you how the disease is transmitted?
8. Yes
9. No
10. Do the CAHWs give you information on prevention?
11. Yes
12. No

**Technical ability**

1. Do the CAHWs examine the animals before treatment?
2. Yes
3. No

**Follow up visits**

1. Do the CAHWs carry out follow up visits after treatment?
2. Yes
3. No

**Types of records kept**

1. Do CAHWs have treatment books?
2. Yes
3. No
4. Do they write any clinical notes in the treatment book
5. Yes
6. No

**Cost of treatment**

1. Are you satisfied with the cost of treatment?
2. Very satisfied
3. Satisfied
4. Somehow satisfied
5. Not satisfied

**SECTION B: DISEASE SURVEILLANCE**

**Participation in community mobilization and sensitization**

1. Do CAHWs hold farmers meetings?
2. Often times
3. Some times
4. Rarely
5. Never
6. Mention the type of information delivered in the meetings
7. Key disease events in the area
8. Diseases events in neighboring areas
9. Planned activities
10. Reports of finished activities
11. Animal Movements
12. Other (specify)______________________________________

**Periodic visits to Kraals/farms**

1. Do CAHWs Visit Kraals when not invited
2. Often times
3. Some times
4. Rarely
5. Never
6. Mention activities done by CAHWs on such visits
7. Gather information on diseases
8. Collect samples
9. Give advise on disease control
10. Deliver news of disease events in area
11. Deliver reports of activities
12. Other (specify)__________________________

**Surveillance reports**

1. To whom do you report disease incidences in your animals?
2. Government veterinarians
3. Private vets
4. CAHWs
5. Drug dealers
6. NGOs, CBOs
7. Traditional Healers
8. Do CAHWs inform you of disease outbreaks in other areas?
9. Often times
10. Some times
11. Rarely
12. Never

**SECTION C: CONTROL OF INTERNAL AND EXTERNAL PARASITES**

**Involvement in community mobilization and sensitization for internal and external parasite control**

1. Which are the common parasites affecting your animals
2. Ticks
3. Mites
4. Worms
5. Flies
6. Other (specify)______________________________________
7. Do you receive any advise on their control?
8. Yes
9. No
10. Who gives this advice?
11. Government veterinarians
12. Private vets
13. CAHWs
14. Drug dealers
15. NGOs, CBOs
16. Traditional Healers
17. How is this advice given to farmers?
18. Community meetings
19. Community outreaches
20. During visit by farmer to Drug shops
21. During casual interactions with CAHWs
22. During farm/kraal visits by CAHWs
23. Other (specify)______________________________________
24. How often do you receive this advice?
25. Often times
26. Some times
27. Rarely
28. Never

**Involvement in routine spraying of animals**

1. How do you control external parasites?
2. Hand picking
3. Spraying
4. Dipping
5. Pour on
6. Other (specify)____________________________
7. What role do CAHWs play in this exercise?
8. Provide acaricides
9. Advise on dilution
10. Supervise application of acaricides
11. Collecting information on activity
12. Sensitization on external parasite control
13. Other (specify)________________________________
14. How often do they get involved?
15. Often times
16. Some times
17. Rarely
18. Never

**SECTION D: SUPPORT TO LIVESTOCK PRODUCTION**

**Ability to demonstrate the best modes of production**

1. Does your CAHW own any animals?
2. Yes
3. No
4. Not aware
5. What kind of bulls do you keep for mating?
6. Strong
7. Active
8. Healthy
9. Fast growing
10. Other (specify)
11. Does the CAHW assist you to select the right bulls for mating?
12. Yes
13. No
14. Do you visit the CAHW and learn from their kraal/farms?
15. Often times
16. Some times
17. Rarely
18. Never

**Ability to advise farmers on animal production**

1. Do CAHWs give you advice about animal production?
2. Often times
3. Some times
4. Rarely
5. Never
6. Besides natural grazing what else do your animals feed on
7. Acacia pods
8. Mineral licks
9. Crop residue
10. Brewers waste
11. Domestic refuse
12. Does your CAHW advise you on alternative feeding?
13. Yes
14. No

**SECTION E: REPORTING**

**Advice to farmers about diseases**

1. Do CAHWs inform you about contagious disease in the district
2. Often times
3. Some times
4. Rarely
5. Never
6. Do CAHWs inform you about the analysis results after samples were taken from your farm?
7. Often times
8. Some times
9. Rarely
10. Never
11. When faced with livestock health challenges who do you call?
12. Government veterinarians
13. Private vets
14. CAHWs
15. Drug dealers
16. NGOs, CBOs
17. Traditional Healers

**SECTION F: VACCINATION**

**CAHWs involvement in official vaccination campaigns**

1. Does the CAHW inform you about the benefits and advantages of vaccination?
2. Often times
3. Some times
4. Rarely
5. Never
6. Does the CAHW give advice on the care of animals post vaccination?
7. Often times
8. Some times
9. Rarely
10. Never
11. Which of the following responsibilities regarding vaccination does the CAHW inform you about?
    1. Cost sharing
    2. Selection of the right age for vaccination
    3. Crush construction
    4. Contra indications for vaccination

**SECTION F: DEHORNING AND CASTRATION**

**Involvement in Dehorning and Castration**

1. Do you de-horn or castrate your animals?
2. Yes
3. No
4. If there is a need for dehorning or castration whom do you call?
5. Government veterinarians
6. Private vets
7. CAHWs
8. NGOs, CBOs
9. Traditional Healers
10. Does the CAHW give you advise on post castration/dehorning care?
11. Often times
12. Some times
13. Rarely
14. Never
15. Do they charge you for castration and dehorning?
16. Yes
17. No
18. Are you satisfied with the cost of dehorning or castration?
19. Very satisfied
20. Satisfied
21. Somehow satisfied
22. Not satisfied

**SECTION G: ANIMAL IDENTIFICATION/BRANDING**

**Involvement in animal identification/branding**

1. Do you brand /identify your animals?
2. Yes
3. No
4. Is the branding/identification a private or an official arrangement?
5. Official
6. Private
7. Who does the branding/identification?
8. Government veterinarians
9. Private vets
10. CAHWs
11. NGOs, CBOs
12. Traditional Healers

**THANK YOU VERY MUCH FOR YOUR TIME**
