# Supplementary material for: Participatory evaluation of delivery of animal health care services by community animal health workers in Karamoja region of Uganda
Source: PLoS One. 2017 Jun 8;12(6):e0179110. doi: 10.1371/journal.pone.0179110 (PMC5464622; doi:10.1371/journal.pone.0179110)
Supplement: S3 Text — (DOCX) [file pone.0179110.s009.docx]

**EVALUATION OF PERFORMANCE OF CAHWS IN KARAMOJA REGION - UGANDA**

**CAHWS QUESTIONNAIRE**

**DISTRICT……………………………………DATE ………………………………**

**QUESTIONNAIRE NO……………….**

**Section A: Treatment**

**Technical ability**

1. Identify common diseases in the locality………………………………………
2. Give three main clinical signs of three common diseases in your area

i………………………..ii………………………..iii……………………..

1. What is the treatment of each disease you have mentioned above

i………………………ii………………………………iii……………………..

**Types of records kept**

1. Mention the types of records kept

………………………………………………………………………………

1. Mention the uses of these records

……………………………………………………………………………….

1. Who uses these records?

………………………………………………………………………………..

**Follow up visits**

1. Do you do follow up visits?

a) Yes b) No

1. If yes what do you do during those visits?

………………………………………………………………………………..

**Type of information provided to farmers**

1. Mention the type of information given to farmers during your visits

………………………………………………………………………………..

**Section B: Disease surveillance**

**Ability to identify common diseases**

1. List the diseases commonly reported in your area?

……………………………………………………………………………….

1. List 4 notifeable diseases you know

……………………………………………………………………………….

1. Do you participate in sample collection?

a) Yes b) No

1. If Yes what type of samples do you collect?
   1. Fecal
   2. Blood
   3. Tsetse flies from traps

**Periodic visits to Kraals/farms**

1. List other reasons for visiting kraals other than treatment

……………………………………………………………………………….

1. Who facilitates these visits?

…………………………………………………………………………………

1. How often do you receive this facilitation?

……………………………………………………………………………………..

**Surveillance Reports**

1. Identify the type of information do you include in the report
2. Number of animal treated
3. Number of animals sick
4. Number of animals dead
5. Location
6. No survived
7. Type of disease
8. Disease infrastructure
9. List the type of feedback given to farmers?

……………………………………………………………………………..

1. Identify the methods you use to give feedback to farmers

……………………………………………………………………………..

**Section C: Control of external parasites**

**Involvement in routine spraying of animals**

1. List the activities you carry out related to spraying of animals

……………………………………………………………………………..

1. Are there official facilities where you organize these activities

a) Yes b) No

1. Name these facilities (e.g. communal crushes, spray races, dips)
2. How are these activities facilitated?

…………………………………………………………………………….

1. Are the drugs readily available

a) Yes b) No

**Demonstrations through kraal out reaches**

1. How many outreaches can you perform in a month?

……………………………………………………………………………

1. List the items required for these outreach demonstrations?

……………………………………………………………………………

1. Who provides these items?

…………………………………………………………………………….

1. What exactly do you do during these outreaches?

……………………………………………………………………………..

**Technical ability**

1. Mention common acaricides and their classes

……………………………………………………………………………..

1. Mention the mode of application of the acaricides

……………………………………………………………………………….

1. What is the dilution rate

…………………………………………………………………………….

**Section D: Livestock Production**

**Ability to advise farmers on animal production**

1. Whether CAHW keeps animals and what type
2. Yes b) No

If yes what type……………………………………………………………..

1. What livestock management practices do you implement?
2. Breeding,
3. Feeding,
4. Housing

**Section F: Reporting**

**Knowledge about official diseases to report**

1. What are the four diseases that you have to report?

……………………………………………………………………………..

**Section G: Vaccination**

**The CAHWs technical abilities**

1. **Mention 3 diseases that can be prevented by vaccination**
   1. PPR
   2. CBPP
   3. FMD
   4. NCD
   5. LSD
   6. Other (specify)…………………………………………….
2. How do you store and manage your vaccines?
   1. Fridges
   2. Cool boxes
   3. Ice packs
   4. Ice boxes
   5. Tree shades
3. List 5 requirements for a successful vaccination exercise

…………………………………………………………………………….

1. How determine the dosage for vaccination

……………………………………………………………………………..

1. What are the vaccination sites for the common vaccines used in Karamoja

……………………………………………………………………………

**Section H: Dehorning and castration**

**Technical ability**

1. List the some of the requirements you need for dehorning and castration

……………………………………………………………………………

1. Mention 2 reasons for dehorning

……………………………………………………………………………

1. Mention the methods of dehorning, castration you use

…………………………………………………………………………..

1. Mention 3 reasons for castration

……………………………………………………………………………..

1. Mention 2 reasons for hoof trimming

……………………………………………………………………………..

1. Mention the type of advise you give to farmers post castration dehorning

**…………………………………………………………………………….**

1. What problems do you encounter dung minor surgery

……………………………………………………………………………….

**Section I: Animal Identification/Branding**

**Technical ability**

1. Mention 3 types of animal identification you normally use

………………………………………………………………………………..

1. What information do the brands/other identification carry?

………………………………………………………………………………….

1. Mention 3 uses of branding /identification

………………………………………………………………………………………..

**Section J: Sustainability**

**The CAHWs volume of activity**

1. How would you classify your volume of activity as a CAHW?

………………………………………………………………………………..

**THANK YOU VERY MUCH FOR YOUR TIME AND COMMITMENT**
