# Supplementary material for: Participatory evaluation of delivery of animal health care services by community animal health workers in Karamoja region of Uganda
Source: PLoS One. 2017 Jun 8;12(6):e0179110. doi: 10.1371/journal.pone.0179110 (PMC5464622; doi:10.1371/journal.pone.0179110)
Supplement: S4 Text — (DOCX) [file pone.0179110.s010.docx]

**EVALUATION OF PERFORMANCE OF CAHWS IN KARAMOJA REGION - UGANDA**

**DVO’s QUESTIONNAIRE**

**DISTRICT……………………………………DATE ………………………………**

**Tel Contact of Respondent________________________Email_________________**

**SECTION A: TREATMENT**

**Technical ability**

1. What criteria did you use to select CAHWs?
   1. Academic qualification
   2. Residence in area
   3. Level of activity
   4. Livestock ownership
   5. Others (specified)……………..
2. What training have they acquired?
3. _______________________________
4. ________________________________
5. ________________________________
6. _________________________________
7. _________________________________
8. ________________________________
9. What is your level of satisfaction regarding their function of treatment?
   1. Excellent
   2. Very good
   3. Good
   4. Fair
   5. Poor
10. How often do you hold support supervision meetings/ field visits?
11. Weekly
12. Bi weekly
13. Monthly
14. Quarterly
15. Other (specify)……………………………
16. At this point in time what is your opinion on the relevance of CAHWs in your district
17. Very relevant
18. Relevant
19. Irrelevant
20. Do you conduct refresher trainings?
21. Yes
22. No
23. If yes how many refresher trainings have you conducted in the last 3 years?__________________
24. If no why?_________________________________________________________

**SECTION B: DISEASE SURVEILLANCE**

**Surveillance Reports**

What disease out breaks have you experienced in the last 5 years?

1. ______________________________
2. _______________________________
3. ______________________________
4. ______________________________
5. _____________________________
6. _____________________________

Did CAHWs report any of these diseases?

1. Yes
2. No
3. How long did they take to report outbreaks?
4. 12 – 24 hours
5. 2 - 7 days
6. 2- 3 weeks
7. More than a month
8. What form of reports?
9. Verbal,
10. Written,
11. Email
12. SMS
13. Calls
14. Others (specify)_____________
15. Level of satisfaction with reports
    1. Very satisfied
    2. Satisfied
    3. Somehow
    4. Not satisfied
16. Is the data provided reflected in the monthly Epidemiological report of MAAIF
    1. Yes
    2. No
17. List interventions you have ever implemented on the basis of these reports
18. ________________________
19. ________________________
20. ________________________
21. ________________________
22. ________________________
23. ________________________

**Support to surveillance**

1. Are CAHWs catered for in the budget for disease surveillance?
2. Yes
3. No
4. Other than financial support name the other form of support you have extended to the CAHWs.
5. ____________________________
6. ____________________________
7. ____________________________
8. ____________________________

**SECTION C: REPORTING**

**CAHWs involvement in reporting**

1. Are CAHWs regularly invited to meetings?
   1. Yes
   2. No
2. Do the CAHW follow DVOs recommendations after outbreak?
3. All the time
4. Most of the time
5. Some times
6. Rarely
7. Not at all
8. Do CAHWs report disease occurrence in less than 12 hours?
9. All the time
10. Most of the time
11. Some times
12. Rarely
13. Not at all
14. Do you receive reports from CAHWs on the following; (Tick as appropriate)
15. Animal movements for Trade
16. Animal movements for Migrations
17. Animal movements for social cultural practices (e.g. marriage or dowry)
18. Census

**SECTION D: VACCINATION**

**CAHWs involvement in official vaccination campaigns**

1. Do CAHWs participate in selection of vaccination sites?
2. Yes
3. No
4. List the diseases that CAHWs have vaccinated against
   1. PPR
   2. NCD
   3. CBPP
   4. FMD
   5. LSD
   6. BR,
   7. Other (specify)
5. Do you receive reports of each vaccination campaign from the CAHWs?
   1. Yes
   2. No

**The setting up of campaigns outside official ones**

1. Does the CAHW carry out vaccination in the framework of government or NGO campaign
2. Government sponsored
3. NGO sponsored
4. Both

**SECTION E: ANIMAL IDENTIFICATION/BRANDING**

**Involvement in animal identification/branding**

1. Are CAHWs involved in branding/Identification of animals?
   1. Yes
   2. No
2. Is the branding/identification a private or an official arrangement?
3. Official arrangement
4. Private arrangement
5. Both
6. Who facilitates these exercises
7. __________________________
8. __________________________
9. ___________________________
10. ___________________________
11. How often do CAHWs provide reports concerning these exercises?
12. Regularly
13. Occasionally
14. Never

**SECTION F: SUSTAINABILITY**

**Support given to the CAHW from his/her professional environment**

1. What kind of support do you provide to CAHWs
2. _______________________________
3. ________________________________
4. ________________________________
5. _________________________________
6. _________________________________
7. Overall are you satisfied with the performance of CAHWs
8. Very satisfied
9. Satisfied
10. Somehow
11. Not satisfied

**THANK YOU VERY MUCH FOR YOUR TIME AND COMMITMENT**
